# Exploring the potential of the ketogenic diet in autism spectrum disorder: metabolic, genetic, and therapeutic insights

**DOI:** 10.1007/s11011-024-01518-1

**Published:** 2025-01-08

**Authors:** Alexa Schrickel, Jop Groeneweg, Eline Dekeyster

**Affiliations:** 1 Institute of Psychology, Leiden University, Wassenaarseweg 52, Leiden, 2333 AK The Netherlands; 2 Faculty of Technology, Policy and Management, Delft University of Technology, Jaffalaan 5, Delft, 2628 BX The Netherlands

**Keywords:** Autism spectrum disorder, Ketogenic diet, Metabolic psychiatry

## Abstract

Current treatment approaches for Autism spectrum disorder (ASD) primarily focus on symptom management rather than addressing underlying dysfunctions. The ketogenic diet (KD), a high-fat, low-carbohydrate diet inducing nutritional ketosis, has shown promise in treating epilepsy and may offer therapeutic benefits for ASD by modulating metabolic and neuroprotective pathways. This review examined the potential impact of KD on underlying mechanisms in ASD. While evidence from human studies on underlying mechanisms is limited, animal research has shown a large overlap of mechanisms modulated by KD and dysfunctions in ASD. As such, targeting multiple disrupted pathways at once, KD presents a potential multifaceted treatment approach for ASD. However, more evidence from human studies is needed on the effectiveness of KD in the modulation of underlying dysfunctions in ASD. Additionally, precision medicine approaches could help identify individuals who would benefit most from the intervention, potentially extending its use to other psychiatric conditions with similar metabolic patterns. Consequently, KD interventions might show the potential to induce a drastic paradigm shift in understanding and treating ASD.

## Introduction

Autism spectrum disorder (ASD) is a lifelong neurodevelopmental disorder affecting 1 in 100 individuals worldwide (Salari et al. 2022; Zeidan et al. 2022). ASD is behaviorally defined by deficits in social interaction and communication as well as restricted and repetitive behaviors (Hyman et al. 2020). However, ASD commonly presents with various comorbidities, such as epilepsy, gastrointestinal (GI) disorders, and psychiatric disorders (Casanova et al. 2020; Doshi-Velez et al. 2014). Some of these comorbidities may be partially explained by genes associated with ASD which have pleiotropic effects on other disorders via the genetic influences on diverse biological pathways (Khachadourian et al. 2023). This genetic complexity, further represented by the heterogeneity of the disorder, also complicates the development of treatment approaches for ASD.

Despite the broad implications of ASD, current treatment effects are limited. Traditional approaches focus on behavioral interventions, e.g. behavioral therapy to target the core symptoms of ASD, and speech and language therapy to improve articulation and communication, whose efficacy is highest when applied early during development (Hyman et al. 2020). Additionally, psychopharmacological approaches have been focused on symptom reduction and management of comorbidities rather than targeting underlying dysfunctions while inducing diverse side effects, many of which are metabolic (Aishworiya et al. 2022).

Individuals with ASD have a reduced quality of life (QoL) compared to individuals without ASD across their lifespan, and while symptoms of ASD show improvements with age, QoL generally remains relatively low (van Heijst and Geurts 2015). Lifetime social costs are significant for individuals with ASD, with costs of up to $3.6 million per individual in the US (Cakir et al. 2020). Thereby, the manifestation of ASD and its associated care has a significant impact on the individuals, their caretakers, and society.

However, a substantial gap remains for treatment approaches for autism, emphasizing the need for new approaches to not only alleviate symptoms but also modulate a wide range of underlying mechanisms.

### Ketogenic diet intervention

The ketogenic diet (KD) consists of high-fat, low-carbohydrate foods and is designed to induce nutritional ketosis, a metabolic state defined by increased blood ketone levels above 0.5mM. Ketones include a group of molecules, β-hydroxybutyrate (BHB), acetoacetate, and acetone, which are derived from fatty acid oxidization in the liver (Altayyar et al. 2022; Kolb et al. 2021). Following the distribution of ketones throughout the body, including the brain, they are taken up by mitochondria to be utilized for energy production. There, they are metabolized into acetyl-CoA and enter the citric acid cycle (Romano et al. 2017). As such, ketosis has diverse effects on overall mitochondrial function, resulting in increased adenosine triphosphate (ATP) levels. That is, ketone bodies are hypothesized to have a higher efficiency as an energy source, resulting in a higher ATP yield per unit of oxygen from ketone bodies compared to glucose (Hartman et al. 2007). Furthermore, the effects of ketosis extend beyond the energy metabolism, influencing multiple signaling pathways involved in, e.g., neuroprotection and inflammation, primarily modulated by improved mitochondrial function and decreased reactive oxygen species (ROS) production (Milder and Patel 2012; Nylen et al. 2009; Vidali et al. 2015).

Ketogenic diet therapy has been successfully implemented as a treatment option for epilepsy for the past decades, thereby indicating a link between metabolic interventions and brain health (Martin-McGill et al. 2020). As such, KD may be implicated in modulating underlying disrupted mechanisms in other disorders, such as ASD, thereby facilitating symptom alleviation.

This review aims to explore the neuroprotective effects of KD in ASD. By addressing underlying mechanisms and, consequently, symptoms, we aim to pave the way for more effective interventions.

## Main/Results

### Ketogenic diet interventions for ASD in humans

So far, the effects of KD on individuals with ASD have been focused on behavioral outcomes rather than the modulation of underlying mechanisms. Evidence from human studies indicated that KD overall, compared to other dietary interventions, has shown to be beneficial for individuals with ASD, especially for symptom reduction of anxiety, attention, cognition, constipation, depression, language/communication, lethargy, seizures, social interactions, and understanding (Matthews and Adams 2023). Furthermore, KD was accompanied by fewer adverse effects than traditional psychiatric/seizure medication (Coleman et al. 2019; Matthews and Adams 2023).

KD interventions include different versions of ketogenic diets, defined by e.g., different macronutrient ratios or additional supplements, which have been shown to reduce core symptoms of ASD in children (Evangeliou et al. 2003; Herbert and Buckley 2013; Spilioti et al. 2013). As such, the Modified Atkins Diet (MAD) specifically improved cognition and sociability, in addition to alleviating overall symptoms (El-Rashidy et al. 2017). Similar improvements in social affect but not restrictive and repetitive behaviors have been observed using a modified KD supplemented with medium-chain triglyceride (MCT) oils (Lee et al. 2018). However, large variability in the diets and use of control measures, i.e. ketone level measurements, used in the current literature impedes insights into the effects of ketone bodies on ASD. The combination of a classic KD with e.g., gluten-free and casein-free diets further complicates the dissection of the impact of ketone bodies from other dietary specifications.

Building on earlier studies, such as Evangeliou et al. (2003), which reported the metabolic effects of KD in ASD patients, Mu et al. (2020) have been one of the first to investigate the underlying mechanisms of KD treatment in humans, providing critical insights into its metabolic pathways. That is, metabolic profiles were assessed in individuals with ASD compared to controls pre- and post-KD intervention, and results indicate distinct compositions of metabolic profiles. Following a modified KD intervention, metabolic phenotypes of ASD have been significantly altered, specifically metabolites and trace elements involved in mitochondrial metabolism, which, in turn, are accompanied by behavioral improvements (Mu et al. 2020).

While KD seems to promote the reduction of behaviorally appearing symptoms of ASD, the number of published evidence remains limited. The existing evidence is based on case studies and studies with small sample sizes ranging from one to 45 participants (Table 1). Furthermore, the type of KD diet differed between the described studies, and the potential differences in the effects of different diets on each individual remain unclear. While this does not necessarily impact the effects of the dietary intervention itself, the overall feasibility of adopting the new diet, as well as long-term adherence, might differ. That is, food selectivity puts limits to the variety of foods that are accepted by individuals with ASD, especially children, posing potential challenges to adopting a new diet (Esposito et al. 2023). Moreover, the overall feasibility of adopting and adhering to KD interventions heavily depends on individuals and their caregivers. That is, ASD severity, related to smell, texture, and taste hypersensitivities, as well as the additional time and labor burden for the caregiver, indicate the success of the diet adoption and adherence (Albers et al. 2023).

**Table 1:** Evidence from human studies

Publication		Tested condition	Age	N (f/m)	Diet	Intervention duration	Outcome measures	Results
Pure KD interventions								
El-Rashidy et al. (2017)		ASD	3–8 years	45 (12/33)	MAD (60:30:10)^a^	6 months	ATEC, CARS	Significant improvements in CARS and ATEC Higher scores in cognition and sociability than GFCF control
Evangeliou et al. (2003)		ASD	4–10 years	30 (14/16)	KD based on John Radcliffe (71:10:19)^a^	6 months	CARS	Significant CARS improvement for 18/30 participants
Matthew and Adams (2023)		ASD	-	21	KD (unspecified)	-	Survey scores for benefits and adverse effects of diet	KD has highest overall benefits for symptoms incl. anxiety, attention, cognition, constipation, depression, language/communication, lethargy, seizures, social interactions & understanding KD has highest adverse effects but lower than psychiatric/seizure medication
Spilioti et al. (2013)		ASD	4–14 years	6	KD (unspecified)	6 months	CARS	Significant improvement of all symptoms
Żarnowska et al. (2018)		ASD + mental retardation + ADHD	6 years	1 (0/1)	KD (2:1)^b^	12 months	^18^FDG-PET, CARS	Decreased ^18^FDG uptake across the whole cortex Overall CARS improvement
Combined KD interventions								
Herbert and Buckley (2013)		ASD + epilepsy + obesity	12 years	1 (1/0)	KD + GFCF (1.5:1)^b^	14 months	CARS	Overall improvement of CARS, social skills, cognition and language, resolution of stereotypies Reduction of seizures for comorbid epilepsy
Mu et al. (2020)		ASD	2–21 years	17 (2/15)	Modified KD + GF + MCT oil^c^	3 months	ADOS-2, CARS-2, GC-MS, H NMR, ICP-MS	Distinct metabolite compositions of control vs. ASD at baseline KD alters metabolic phenotype of ASD for metabolites and trace elements involved in mitochondrial metabolism
Lee et al. (2018)		ASD	2–17 years	15 (2/13)	Modified KD + GF + MCT oil^c^	3 months	ADOS-2, CARS-2	Significant improvements in social affect score and overall score No significant improvements in restricted and repetitive behaviors

*Note. ASD* Autism Spectrum disorder, *MAD* Modified Atkins Diet, *ATEC* Autism Treatment Evaluation Test, *CARS* Childhood Autism Rating Scale, *GFCF* Gluten-free Casein-free diet, *KD* Ketogenic Diet, *ADHD* Attention Deficit Hyperactivity Disorder, ^*18*^*FDG-PET* 18 Fluoro-Deoxyglucose Positron Emission Tomography, *GF* Gluten-free, *MCT* Medium-Chain Triglyceride, *ADOS-2* Autism Diagnostic Observation Scale 2, *GC-MS* Gas Chromatography-Mass Spectrometry, ^*1*^*H NMR MS*^1^H Nuclear Magnetic Resonance Spectroscopy, *ICP-MS* Inductively Coupled Plasma-Mass Spectroscopy

^a^ Dietary composition in percentages (fat:protein:carbohydrates)

^b^ Dietary composition as a ratio (fats:proteins+carbohydrates)

^c^ 20% of energy requirements covered by MCT oil, carbohydrates limited to 20–25 g/day

### Underlying mechanisms

#### Brain energy metabolism

The brain is a highly metabolically active organ, continuously converting glucose and fats into utilizable energy to enable complex functions and maintain its structure (Rolfe and Brown 1997). Metabolic dysfunction in ASD is indicated by elevated levels of the molecular byproducts, lactate and pyruvate, of the glucose energy metabolisms. Elevated levels of these metabolic byproducts have been used as biomarkers of mitochondrial dysfunction and, thereby, decreased energy production and availability for cells (Rossignol and Frye 2012). Additionally, deficits in energy metabolism are further observed in the form of decreased expression of the electron transport chain (ETC) complexes in the cerebellum and frontal and temporal regions of the brain (Chauhan et al. 2011; Weissman et al. 2008). These mitochondrial function abnormalities have been linked to ASD-related clinical symptoms, such as repetitive behaviors, social deficits, and hyperactivity (Rossignol and Frye 2012). Furthermore, alterations of cerebral glucose metabolisms have been shown in individuals with ASD. That is, glucose hypometabolisms have been identified in the cerebellum and anterior temporal cortices, while hypermetabolisms occur in the bilateral frontal cortices (Kumar et al. 2017). The altered utilization of glucose is further suggested by the genetic overlap between neurodevelopmental disorders and metabolic syndrome (MetS), however, their association with ASD specifically remains suggested but unproven (Fanelli et al. 2022). A key feature of MetS is insulin resistance (IR), which occurs when cells do not respond adequately to insulin, a hormone produced by the pancreas that regulates blood sugar levels. In turn, IR can lead to elevated glucose levels as well as disruptions in insulin signaling, which in the CNS is implicated in synaptic plasticity, neurotransmission, apoptosis, and neuroinflammation (Fanelli et al. 2022; Stern 2011).

In general, KD offers an alternative energy supply via the utilization of ketones by mitochondria, thereby boosting the availability of ATP. Energy availability has a bidirectional relationship with mitochondrial function (Ahn et al. 2020; Newell et al. 2016). That is, KD has been found to improve mitochondrial function and morphology in a mouse model of ASD (Ahn et al. 2020). Following a two-week KD, the mean length of mitochondria in a mouse model of ASD increased, and fragmentation of mitochondria was restored, which are both linked with improved ATP production and ROS, thereby potentially reversing ASD-like etiologies as shown in animal models (Ahn et al. 2020). Additionally, mitochondrial biogenesis, in general, is improved via altered mRNA and protein expression following KD (Hasan-Olive et al. 2019).

#### GABA/Glutamate function

While glutamate is the primary excitatory neurotransmitter, γ-aminobutyric acid (GABA) is the main inhibitory neurotransmitter in the central nervous system (CNS), crucial for the regulation of neural activity (Sears and Hewett 2021). The imbalance between these excitatory/inhibitory neurotransmitters has been recognized as a prominent feature of ASD and has been linked to repetitive behaviors and anxiety in children with ASD (Choudhury et al. 2012; Zhao et al. 2022). However, these neurotransmitter pathologies go beyond signal transmission, playing important roles in brain maturation and cortical reorganization and, as such, present a crucial aspect of neurodevelopment (Choudhury et al. 2012).

Following a KD has been shown to regulate this imbalance by inhibiting glutamate transporters, thereby correcting the ratio between excitatory and inhibitory neurotransmitters (Lutas and Yellen 2013; Romano et al. 2017). Additionally, ketones level the GABA/glutamate ratio by acting as a substrate during glutamate production in inhibitory presynaptic terminals, thereby increasing the synthesis of GABA via the breakdown of glutamate by glutamate decarboxylase (GAD; Rho and Boison 2022). BHB, but not other ketone bodies, inhibits histone deacetylase 1 and 2 complexes (HDAC1/HDAC2), which upregulates sirtuin 4 (SIRT4) and GAD1 gene transcription via open chromatin. Consequently, these mechanisms increase the availability of GABA in the brain and further regulate the GABA/glutamate ratio (Calderón et al. 2017; Qiao et al. 2024). Additionally, Grin2A and Grin2B genes encode subunits of NMDA receptors, which are a type of ionotropic glutamate receptor. In ASD, postmortem tissue analysis showed that histone acetylation is significantly lower. However, KD interventions elevate the histone acetylation, which increases the translation of the NMDAR subunit genes in the PFC, thereby lessening social deficits associated with an ASD mouse model (Qin et al. 2022). As such, the modulation of neurotransmitter availability in various areas of the CNS might potentially reduce ASD-related behaviors.

These findings highlight the multifaceted nature of ASD pathophysiology and the potential of interventions such as the KD in modulating neurotransmitter systems implicated in the disorder. By targeting GABAergic and glutamatergic pathways, the KD offers a novel approach to restoring synaptic balance and ameliorating core symptoms of ASD, providing valuable insights into the neurobiological mechanisms underlying the disorder.

#### Oxidative stress

Oxidative stress is characterized by an imbalance of prooxidants and antioxidants that results in cell damage (Bjørklund et al. 2020). Oxidative stress has widespread effects, causing changes in lipid peroxidation, protein and DNA oxidation, inflammation, altered immune response, decreased DNA methylation, and epigenetic dysregulation (Bjørklund et al. 2020). Prooxidants/reactive oxygen species (ROS) are signaling molecules produced during the energy production of cells as part of the electron transporter chain. In a mouse model of ASD, the production of oxidative species is increased due to mitochondrial dysfunction, specifically, electron transporter chain irregularities that cause increased leakage of ROS (Bjørklund et al. 2020; Pangrazzi et al. 2020). Additionally, antioxidants are metabolized incorrectly (Pangrazzi et al. 2020). That is, in ASD, the production of glutathione, the most common antioxidant precursor in cells, is impaired, which limits the ability to detoxify ROS (Bjørklund et al. 2020; Milder and Patel 2012).

KD protects against oxidative stress via several routes. The antioxidant metabolism is improved as KD increases the biosynthesis of glutathione via the Nrft2 signaling pathway activation (Milder and Patel 2012). Ketones also decrease the production of ROS by increasing the translation of NADP + to NADPH and inhibiting the transfer of electrons from coenzyme Q (QH_2_) to oxygen at complex 1 of the electron transporter chain (Norwitz et al. 2019). Additionally, ketones regulate ROS by inhibiting histone deacetylase (HDAC), which in turn increases the expression of FOXO3A and MT2, both genes crucial for oxidative stress resistance, thereby further increasing the protection against oxidative stress effects (Shimazu et al. 2013).

#### mTOR regulation

The mammalian target of rapamycin (mTOR) signaling regulates various cellular processes such as cell growth, gene expression, and synaptic functions (Thomas et al. 2023). Specifically, mTOR forms two distinct protein complexes, mTORC1 and mTORC2. mTORC1 responds to nutrient signals, like glucose and amino acids (Thomas et al. 2023). In turn, it controls biological functions, such as energy metabolism, autophagy, and protein synthesis, including mitochondrial metabolism and biogenesis (Laplante and Sabatini 2009). mTORC2 responds to growth factors and is implicated in cell growth and proliferation as well as motility (Thomas et al. 2023). In ASD, irregularities in the activation of mTOR signaling pathways have been observed, specifically, hyperactivation of mTORC1 is hypothesized to be a likely primary driver of ASD symptoms (Thomas et al. 2023; Winden et al. 2018). That is, abnormalities of TSC1, TSC2, and PTEN, which are negative regulators of mTORC1, elicit ASD-like phenotypes in animal models (Sharma and Mehan 2021). Moreover, mitochondrial increased activity due to activated mTORC1 has been reported to contribute to significant mitochondrial loss and ASD-like symptoms in animal models (Sharma and Mehan 2021). Therefore, the direct modulation of mTORC1 specifically has been suggested as a beneficial therapeutic target for ASD.

Although the impact of KD has not been differentiated for mTORC1 and mTORC2 yet, KD has been shown to inhibit general mTOR signaling pathways in the brain, specifically the hippocampus, and the liver in mouse models of ASD, as shown by lowered expression of pS6 and pAkt, indicators of mTOR activation (McDaniel et al. 2011; Winden et al. 2018). Given the link between mTOR pathways and ASD, KD has the potential to have therapeutic effects, thereby rescuing symptoms. That is, pharmacological inhibition of mTOR has been shown to improve social interaction in rat models of ASD (Zhang et al. 2016). However, differentiated research on the effects of KD on mTORC1/2 for ASD is needed to fully understand the altered mechanisms and to work toward specific therapy.

#### Genetics

ASD has an approximate 50–90% heritability (Kreiman and Boles 2020; Sandin et al. 2017; Tick et al. 2016). As such, genetic factors play a fundamental role in ASD as symptomatology arises from polygenic factors, where multiple variants, each with small effects, attribute to the larger cumulative effect (de la Torre-Ubieta et al. 2016). As such, genetic heterogeneity presents challenges when understanding the wide range of etiologies associated with ASD. Genetic variations impact various molecular and cellular mechanisms, with not one uniquely implicated variation responsible but multiple changes to the genetic architecture that result in ASD-like phenotypes in mice (de la Torre-Ubieta et al. 2016). These mechanisms involve genes that impact, e.g., insulin-like growth receptors and glucose transporters, such as the GLUT3 gene responsible for the uptake of glucose into neurons, glutamate transporter genes responsible for synaptic signaling and immune function (Ayhan and Konopka 2019; Zhao et al. 2010). Furthermore, a pleiotropic effect of genetic disruptions has been identified. That is, molecular underpinnings related to ASD transcriptome profiles have been shown to overlap with other neurodevelopmental and psychiatric disorders, many of which are common comorbidities of ASD, thereby explaining the high frequency of diagnosed comorbidities in ASD (Khachadourian et al. 2023).

KD has been specifically shown to affect epigenetic processes, e.g., histone acetylation (An and Claudianos 2016; Qin et al. 2022). BHB specifically can inhibit HDACs, thereby altering gene expression via the modification of chromatin, the carrier of chromosomal DNA (Newman and Verdin 2014, 2017). That is, inhibition of HDACs increases the acetylation of histones and thus relaxes chromatin into an open state. In the open state, the loosened chromatin structure allows access to the transcriptional factors and activators, which promotes gene transcription (Gräff and Tsai 2013). Moreover, gene transcription changes have been identified in the temporal cortex and hippocampal regions, affecting various genes related to cellular stress response and neuronal signaling (Mychasiuk and Rho 2017).

Therefore, it is evident that despite the high heterogeneity of ASD, dysfunctions of various underlying pathways are implicated in the pathology of ASD and rarely act in isolation from each other.

## Future prospects and conclusion

Understanding the ketone metabolism and its effects on underlying mechanisms provides the essential foundation to develop KD-associated treatment approaches. Ketones have been shown to alter underlying mechanisms by acting as metabolites as well as signaling molecules and have thereby been shown to rescue several underlying mechanisms disrupted in ASD.

Despite the promising insights gained from the reviewed animal studies, there has been a significant gap in translating these findings into human research. Since 2003, only a handful of studies have explored KD as a treatment for ASD in humans, few of which investigated the underlying mechanisms of KD in ASD (Evangeliou et al. 2003; Mu et al. 2020). In contrast, numerous investigations have focused on understanding the mechanisms underlying KD interventions in rodent models of ASD. This highlights a crucial need for more comprehensive research to bridge the gap between preclinical findings and clinical applications, ensuring that the potential benefits of KD for ASD can be fully understood and effectively utilized in humans.

That is, given the high heterogeneity of ASD manifestations in individuals, it is unlikely that all patients will exhibit a uniform response to KD, and some may not respond at all. Therefore, identifying biomarkers and subgroups within the ASD population is crucial to identifying potential high responders to this treatment approach. A deeper understanding of metabolic and genetic variables could significantly enhance our ability to estimate the likelihood of success for specific subgroups. As such, the role of precision medicine is to maximize patient outcomes by individualizing treatment to a broad range of patient features such as genome profiling, drug response, disease development period, physical states, and causal interferences (Liu et al. 2024). Accordingly, a simple, standardized approach is unlikely to be effective, however, optimizing the diets adaptability may increase the likelihood of effective outcomes in certain subgroups of the ASD population. Thus, future studies should aim to gather extensive data to identify specific subgroups, using large-scale research approaches that incorporate genetic and metabolic markers, to work towards precision medicine and outcome prediction.

Furthermore, regardless of the precision adaptability and outcome of the intervention, a KD intervention may not be feasible for all individuals with ASD. That is, ASD-specific challenges, such as food selectivity and feeding difficulties particularly displayed in children, may hinder successful diet adoption and adherence (Esposito et al. 2023; Albers et al. 2023). Therefore, future studies should also address the feasibility of KD in ASD, examining not only its long-term effects but also its practicality within the context of the behavioral challenges unique to ASD.

In conclusion, while KD holds great potential for regulating underlying mechanisms of ASD, current evidence is limited by small sample sizes, varying protocols, and insufficient data to identify the most effective versions of KD for targeting the described metabolic pathways. There is a clear need for rigorous, large-scale studies to address these limitations and evaluate KD’s sustained effects, feasibility, and impact on ASD mechanisms. By bridging preclinical findings with human research and applying precision approaches, KD can be better understood and potentially utilized as an effective treatment modality in ASD.

## Data Availability

No datasets were generated or analysed during the current study.
